# Cover Crop Management Practices Rather Than Composition of Cover Crop Mixtures Affect Bacterial Communities in No-Till Agroecosystems

**DOI:** 10.3389/fmicb.2019.01618

**Published:** 2019-07-09

**Authors:** Sana Romdhane, Aymé Spor, Hugues Busset, Laurent Falchetto, Juliette Martin, Florian Bizouard, David Bru, Marie-Christine Breuil, Laurent Philippot, Stéphane Cordeau

**Affiliations:** ^1^Université Bourgogne Franche-Comté, INRA, AgroSup Dijon, Agroécologie, Dijon, France; ^2^INRA, UE115 Domaine Expérimental d’Epoisses, Dijon, France

**Keywords:** cover crops, conservation agriculture, bacterial diversity, nitrogen cycling, denitrifiers, nitrifiers

## Abstract

Cover cropping plays a key role in the maintenance of arable soil health and the enhancement of agroecosystem services. However, our understanding of how cover crop management impacts soil microbial communities and how these interactions might affect soil nutrient cycling is still limited. Here, we studied the impact of four cover crop mixtures varying in species richness and functional diversity, three cover crop termination strategies (i.e., frost, rolling, and glyphosate) and two levels of irrigation at the cover crop sowing on soil nitrogen and carbon dynamics, soil microbial diversity, and structure as well as the abundance of total bacteria, archaea, and *N*-cycling microbial guilds. We found that total nitrogen and soil organic carbon were higher when cover crops were killed by frost compared to rolling and glyphosate termination treatments, while cover crop biomass was positively correlated to soil carbon and C:N ratio. Modifications of soil properties due to cover crop management rather than the composition of cover crop mixtures were related to changes in the abundance of ammonia oxidizers and denitrifiers, while there was no effect on the total bacterial abundance. Unraveling the underlying processes by which cover crop management shapes soil physico-chemical properties and bacterial communities is of importance to help selecting optimized agricultural practices for sustainable farming systems.

## Introduction

Agriculture faces several challenges to improve crop productivity, while ensuring ecosystem sustainability. Over the years, there has been increasing concerns about the negative impact of conventional agricultural practices such as monoculture cropping, tillage, pesticides, and fertilizers on the environment with water and soil pollution, greenhouse gas emissions, and decrease of biodiversity (Tilman et al., 2001). Hence, cover cropping, an old management practice, is now emerging as a challenging agricultural practice that can play a major role in enhancing sustainable agriculture and supporting ecosystem services (Schipanski et al., 2014; Groff, 2015). Cover crops can suppress weeds by competing for light, water, and nutrients (Brust et al., 2014; Cordeau et al., 2015) or the release of allelopathic exudates that inhibits weeds (Weston, 1996). Ecosystem services provided by cover crops include protecting soils from erosion and reducing soil nutrient losses by leaching and run-off (Kuo and Sainju, 1998). Furthermore, cover crops can modify soil properties by increasing organic matter where there are mixtures with a high C:N ratio, total nitrogen where there are legume-based mixtures, and thus in all cases enhancing soil nutrients available for succeeding crops (Hubbard et al., 2013). A recent study also reported that cover cropping has positive effects on plant production leading to an increase in crop yields up to 24% in reduced tillage organic systems but only of 2% in conventional system with tillage (Wittwer et al., 2017).

Plant species identity, plant development stage, and plant community composition are well-known drivers of soil microbial communities (Bardgett and van der Putten, 2014; Schlatter et al., 2015), most likely through the release by plant roots of photosynthates that can be used as carbon sources by soil microorganisms (Philippot et al., 2013). As such, recent works demonstrated that cover crops can have beneficial effects on soil microbial communities (reviewed in Vukicevich et al., 2016). Long-term cover cropping has been shown to increase soil nutrient availability, such as organic C, which strongly stimulated the abundance and the diversity of microbial communities (Schmidt et al., 2018). Furthermore, Chavarria et al. (2016) reported an increase in soil bacterial phospholipid fatty acid and soil enzyme activities of 6.8 and 20%, respectively, under two types of cover crop mixtures. However, while there is a growing interest in cover crops due to their capacity to provide a variety of agroecosystem services (Mendes et al., 2013; Philippot et al., 2013), there is still a paucity of information on how cover crops affect soil microbial communities and their functions especially in comparison to the large body of literature on the influence of various agricultural practices on soil microorganisms (Wortman et al., 2013; Calderon et al., 2016).

In this study, we focused on the impact of the cover crops management practices on the diversity, composition, and functioning of soil bacterial communities because of their central role in nitrogen cycling processes. Here, we hypothesize that cover crop mixtures and management modified soil physico-chemical properties which, in turn, led to changes in soil bacterial composition and abundance with possible consequences on nutrient cycling. In this context, the objective of this study was to investigate the impact of agro-ecosystem diversification through four cover crop mixtures (compared to a bare soil control) conducted with different combinations of practices (i.e., three different cover crops termination and two levels of irrigation) on bacterial communities. For this purpose, we assessed at different dates (i.e., in cover crops, after cover crop termination and in the succeeding crop) the soil physico-chemical properties as well as the diversity of the soil bacterial community using Illumina sequencing of the 16S rRNA gene. The abundance of total bacteria, archaea, and N-cycling-related communities were also quantified using real-time PCR.

## Materials and Methods

### Site Description and Management Practices

This experiment was conducted at the experimental farm INRA-Epoisses, France (47°14′11.2^″^ N 5°05′56.1^″^ E). The climate at the site is oceanic with a semi-continental tendency, characterized by cold winters (average daily temperature of 4°C and average monthly precipitation of 43 mm) and hot summers (average daily temperature of 18°C and average monthly precipitation of 69 mm). The soil texture was 41.9% clay, 51.9% silt, and 6.2% sand. The soil properties were: organic carbon = 15.15 ± 1.32, organic matter = 26.30 ± 2.28, and total nitrogen = 1.40 ± 0.11 g kg^–1^ dry soil. Cropping history comprised winter wheat (*Triticum aestivum* L. cv. Apache) in 2010, maize (*Zea mays* L.) in 2011, spring barley (*Hordeum vulgare* L. cv. Sebastian) in 2012, maize (*Z. mays* L.) in 2013, winter wheat (*T. aestivum* L. cv. Apache) in 2014, sunflower (*Helianthus annuus* L. cv. NK ferti) in 2015, winter wheat (*T. aestivum* L. cv. Rubisko) in 2016 followed by the cover crop trial in 2016–2017. The field was moldboard plowed (25 cm depth) once every 2 years. The number of secondary tillage operations (from 5 to 10 cm depth) varies from 1 to 3 per year. N fertilization for the preceding crop (winter wheat) was minimized (only two N applications of 62 and 41 kg ha^–1^ during wheat growth and no further fertilization from wheat tillering stage to harvest) to ensure a null soil N balance at harvest. The wheat straw was removed from the field and the soil was tilled (10 cm depth) twice before starting the different cover crop management practices. The experiment was set up as a block design divided in three blocks (Supplementary Figure S1), each comprising five randomized cover crop treatments, two levels of irrigation, and three techniques for cover crop termination resulting in a total of 90 plots (10 m^2^ each). Cover crop treatments included 12 different species that were sown in August 2016 as a mixture of two or eight species, with or without legumes [i.e., bare soil control, two species without legume (2 Leg−), two species with legumes (2 Leg+), eight species without legumes (8 Leg−), and eight species with legumes (8 Leg+)] (Supplementary Table S1). Cover crop mixtures were sown following a replacement design (Snaydon, 1991; Hamilton, 1994). The seeding rate of each species was the recommended seeding rate in pure stand divided by two or eight in case of species mix of two or eight species, respectively. Recommended seeding rates were: 30 kg ha^–1^ for buckwheat (*Fagopyrum esculentum* cv. Harpe), 40 kg ha^–1^ for vetch (*Vicia sativa* cv. Nacre), 5 kg ha^–1^ for brown mustard (*Brassica juncea* cv.cv. Vitamine), 10 kg ha^–1^ for niger (*Guizotia abyssinica* cv. Regyne), 25 kg ha^–1^ for berseem clover (*Trifolium alexandrinum* cv. Tabor), 10 kg ha^–1^ for wild turnip (*Brassica rapa* cv. campestris), 10 kg ha^–1^ for lacy phacelia (*Phacelia tanacetifolia*), 20 kg ha^–1^ for spring flaxseed (*Linum usitatissimum* cv. Omegalin), 100 kg ha^–1^ for spring faba bean (*Vicia faba* cv. Irena), 35 kg ha^–1^ for sunn hemp (*Crotalaria juncea*), 80 kg ha^–1^ for rye (*Secale multicaule*), 20 kg ha^–1^ for bristle oat (*Avena strigosa*). The entire field was rolled after the cover crop sowing to improve soil–seed contact and to ensure a fast cover crop emergence. The two levels of irrigation (0 or 40 mm) were applied the day after cover crop sowing. The irrigation was delivered with linear precision sprinklers. Finally, the three different terminations of cover crops were (1) naturally by frost (i.e., winter-kill), (2) mechanically by rolling the cover crops with a roller crimper the first day of frost (−4°C, December 15, 2016), and (3) chemically by application of a mixture of glyphosate 3 L ha^–1^ and 2,4-dichlorophenoxyacetic acid at 0.3 L ha^–1^ doses (hereafter indicated as glyphosate treatment) (February 28, 2017). Buckwheat, vetch, brown mustard, niger, berseem clover, wild turnip, lacy phacelia, spring flaxseed, spring faba bean, and sunn hemp are more sensitive to frost than grass species (i.e., rye and bristle oat) which last over the winter (Brandsaeter and Netland, 1999; Mirsky et al., 2009). Spring barley was directly drilled on March 13, 2017 without any pre-sowing tillage. The spring barley was not fertilized or sprayed with any pesticides during this experiment. The selected management practices and crops are commonly used under this climate in low input and conventional systems.

### Plant Biomass Assessment

The aboveground total biomass (cover crop and weeds) was assessed 92 days after sowing (November 12, 2016) by two 0.25 m^2^ quadrats per plot. Quadrats were located next to the soil sampling cores (see next section). Biomass collected were dried in oven (80°C, 72 h) and weighted. Data collected in both quadrats were then pooled.

### Soil Sampling

Sampling was conducted three times during the experiment. The first sampling (T1) was carried out at maximum biomass of the cover crop and before the first frost (November 8, 2016). The second sampling (T2) was conducted few days before the spring barley sowing and thus after all types of cover crop termination (March 3, 2017). The final sampling (T3) was conducted at the spring barley flowering (July 4, 2017). At each sampling date, two soil cores were collected randomly from each replicated plot using a soil corer of 20 cm depth and 5 cm diameter. Soil cores from each plot were then homogenized into a composite sample and sieved at 2 mm (a total of 270 soil samples). Plant debris and roots were removed. Soil relative moisture was determined by drying 10 g of fresh soil at 105°C for 24 h. The physical and chemical soil characteristics (Supplementary Table S2) were measured for all samples according to ISO 14235 and ISO 13878 standards (SADEF, Société Alsacienne pour le Développement et l’Etude de la Fertilité, pôle d’Aspach, France).

### Quantification of N-Cycling Bacterial and Archaeal Communities

DNA was extracted from 250 mg of each soil sample using the DNeasy PowerSoil-htp 96-well DNA isolation kit (Qiagen, France). Total bacterial community was quantified using 16S rRNA primer-based real-time quantitative PCR (qPCR) assays (Muyzer et al., 1993; Ochsenreiter et al., 2003). The nitrification gene *amoA* and the denitrification genes *nirK* and *nirS* were used as molecular markers to quantify the bacterial and thaumarchaeal ammonia-oxidizing (AOB and AOA, respectively) and the denitrifying communities, as described previously (Leininger et al., 2006; Bru et al., 2011), while the *nosZI* and *nosZII* genes were used to quantify the N_2_O-reducers (Henry et al., 2006; Jones et al., 2013). qPCR reactions were carried out in a ViiA7 (Life Technologies, United States) in a 15 μl reaction volume containing 7.5 μL of Takyon MasterMix (Eurogentec, France), 1 μM of each primer, 250 ng of T4 gene 32 (QBiogene, France), and 1 ng of DNA. Two independent replicates were performed for each real-time PCR assay. Standard curves were obtained using serial dilutions of linearized plasmids containing appropriated cloned targeted genes from bacterial strains or environmental clones. PCR efficiency for the different assays ranged from 89 to 100% except for *nosZ*II (78% in average). No template controls gave null or negligible values. Inhibition in qPCR assay was tested by mixing soil DNA extracts with either control plasmid DNA (pGEM-T Easy Vector, Promega, France) or water. No inhibition was detected in any case.

### Amplicon Generation and MiSeq Sequencing

Amplicons were generated in two steps according to Berry et al. (2011). In the first step, the bacterial 16S rRNA gene V3–V4 hypervariable region was amplified by polymerase chain reaction (PCR) using the fusion primers U341F (5′-CCTACGGGRSG CAGCAG-3′) and 805R (5′-GACTACCAGGGTATCTAAT-3′) (Takahashi et al., 2014), with overhang adapters (forward: TCG TCGGCAGCGTCAGATGTGTATAAGAGACAG, adapter: GTC TCGTGGGCTCGGAGATGTGTATAAGAGACAG) to allow the subsequent addition of multiplexing index-sequences using Illumina Nextera indexes. PCR was carried out in duplicate 15 μL reactions containing 7.5 μL Phusion High-Fidelity PCR Master Mix (Thermo Scientific), 0.25 μM of each primer, 250 ng T4 gp32 (MPBio), and 1 ng template DNA. Thermal cycling conditions were 98°C for 3 min followed by 25 cycles of 98°C for 30 s, 55°C for 30 s, and 72°C for 30 s, with a final extension at 72°C for 10 min. Duplicate first step PCR products were pooled then used as template for the second step PCR. In the second step, PCR amplification added multiplexing index-sequences to the overhang adapters using a unique multiplex primer pair combination for each sample. The reaction was carried out in duplicate 30 μL volumes containing 15 μL Phusion High-Fidelity PCR Master Mix (Thermo Fisher Scientific), 1 μM of one forward and one reverse multiplex primer, and 6 μL of first step PCR product. Thermal cycling conditions were 98°C for 3 min followed by eight cycles of 98°C for 30 s, 55°C for 30 s, and 72°C for 30 s, with a final extension at 72°C for 10 min. Duplicate second step PCR products were pooled then visualized in 2% agarose gel to verify amplification and size of amplicons (around 630 bp). The amplicons were cleaned-up purification using sequalPrep^TM^ Normalization plate kit 96-well (Invitrogen) and followed by equimolar pooling. Sequencing was performed on MiSeq (Illumina, 2 × 250 bp) using the MiSeq reagent kit v2 (500 cycles). Demultiplexing and trimming of Illumina adaptors and barcodes was done with Illumina MiSeq Reporter software (version 2.5.1.3).

### Bioinformatic Analysis of the 16S rRNA

Sequence data were analyzed using an in-house developed Python notebook piping together different bioinformatics tools (available upon request). Briefly, 16S rRNA sequences were assembled using PEAR (Zhang et al., 2014) with default settings. Further quality checks were conducted using the QIIME pipeline (Caporaso et al., 2010b) and short sequences were removed (<400 bp). Reference-based and *de novo* chimera detection as well as clustering in OTUs were performed using VSEARCH (Rognes et al., 2016) and Greengenes’ representative set of 16S rRNA sequences as the reference database. The identity thresholds were set at 97%. Representative sequences for each OTU were aligned using PyNAST (Caporaso et al., 2010a) and a 16S rRNA phylogenetic tree was constructed using FastTree (Price et al., 2010). Taxonomy was assigned using UCLUST (Edgar, 2010) and the latest released greengenes database (v.05/2013; McDonald et al., 2012). α-Diversity metrics and UniFrac distance matrices (Lozupone et al., 2011) were calculated based on rarefied OTU tables (5000 sequences per sample). Raw sequences were deposited at the NCBI under the accession number SRP142624.

### Statistical Analyses

Statistical analyses were conducted using R statistical software version 3.4.1. We used an analysis of variance (ANOVA) model to determine the effects of treatments on soil properties and soil microbial diversity, composition and abundance. Normality and homogeneity of the residuals distribution was inspected and log-transformations were performed when necessary. Non-metric multidimensional scaling (NMDS) of the weighted Unifrac distance matrix was used to visualize the bacterial community structure. The effects of soil properties on the bacterial community were analyzed using the envfit function within the vegan package (Oksanen et al., 2018) and projected into the ordination using arrows. Regularized canonical correlation analysis (rCCA) were performed using the mixOmics package (Gonzalez et al., 2008) between bacterial groups at genus level (OTU counts) and soil properties. Permutational multivariate analysis of variance (PermANOVA) was used to test significant differences in communities’ structure using adonis function implemented in the vegan package (permutations = 1999). Correlations between the total aboveground plant biomass and soil properties during the cover crop period of the experiment (T1) were tested using Pearson correlation coefficients.

## Results and Discussion

### Cover Crop Management Affects Soil Physico-Chemical Properties

**FIGURE 1:**
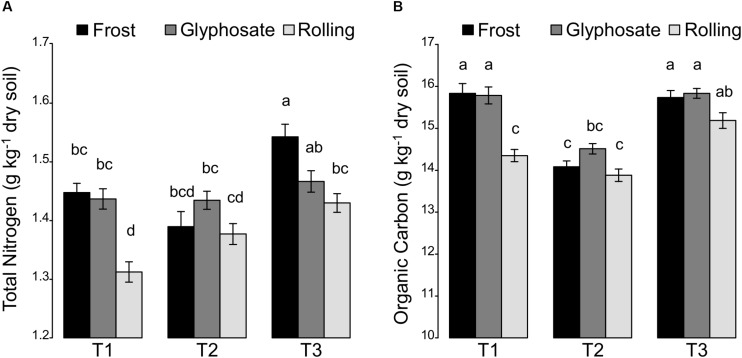
Effects of the interaction between sampling time (T1, T2, and T3) and cover crop termination by frost, glyphosate, or rolling on soil **(A)** total nitrogen and **(B)** organic carbon. All values are means ± standard errors.

Effects of cover crop management (i.e., cover crop mixtures, irrigation, and cover crop termination) on soil physico-chemical properties were analyzed using an ANOVA model (Supplementary Table S3). Since we observed a spatial variability of some soil properties (pH, texture, organic carbon, and total nitrogen) in the experimental field that was not related to the studied treatments, a block effect and an “Y-position in the field” effect were included in our ANOVA model for distinguishing the effects of the studied treatments from those of the spatial structuring of soil properties. Despite this strong spatial variability of soil properties, we did find a strong effect of cover crop management (Supplementary Table S3). Thus, during the cover crop period, the total aboveground plant biomass was positively correlated to both organic C and C:N ratio (*r* = 0.30, *P* < 0.01 and *r* = 0.36, *P* < 0.001, respectively) (Supplementary Figure S2). This correlation might be explained by the release of carbon compounds by cover crops roots (Kuo and Sainju, 1998; Couedel et al., 2018). Before cover crop termination (T1), the differences in total N were related to a block effect rather than to cover crop termination treatments. However, after cover crop termination (T2), we found that the termination treatments lead to a differential shift in the total N (Figure 1A). Thus, the total N soil content remained the same at T2 compared to T1 in the glyphosate treatment. In contrast, the total N content increased regularly for the rolling treatment with about 9.2% increase at T3 compared to T1 (Supplementary Figure S3). In contrast, the frost treatment only stimulated total N content at spring barley flowering (T3). This is consistent with previous studies showing that rolling cover crop enhances N mineralization (Parr et al., 2014; Mirsky et al., 2017). Such differences between the effects of the rolling and frost termination treatment are likely due to the fact that rolling terminated cover crops efficiently and early, while frost treatment killed cover species in succession according to winter hardiness and glyphosate treatment terminated all cover crop species at the same time but at a late stage (early March). While it can be hypothesized that (i) legume-based mixtures take up less nitrogen than non-legume-based mixtures and/or (ii) mixtures with nitrophilic *Brassicaceae* species take up more nitrogen, no significant changes in N were observed between the different cover crop mixtures (Supplementary Table S3). This is in contrast to In’t Zandt et al. (2018) reporting an effect of cover crop mixture on nitrate contents in soil. Overall, our results suggest that frost and rolling but not glyphosate termination method can lead to increase of the total N in soil.

A temporal dynamic of organic C was also observed (Figure 1B) with a decrease in soil organic C between T1 and T2 that was less marked in the glyphosate cover crop termination treatment than in the two other treatments. However, levels of soil organic C were recovered by the flowering of spring barley crop (T3), and even increased in the rolling treatment compared to T1. These results confirm the importance of cover crop termination for the decomposition of cover crop residues as observed for the total N (Van Meeteren et al., 2007).

Interestingly, we found that the soil pH was significantly influenced by irrigation and sampling time (ANOVA, *P* < 0.05, Supplementary Table S3). The soil pH decreased between T1 and T2, and then remained constant at about 7.06 between T2 and T3. We also observed a significantly lower pH in the plots that were irrigated at cover crop sowing compared to the non-irrigated plots, and that last over time (no sampling time-by-irrigation effect detected). Altogether our results indicate that cover crop management, especially cover crop termination and irrigation in interaction with the sampling time, rather than cover crop mixture have a significant impact on soil properties.

### Shifts in Bacterial Community Reflect Differences in Soil Properties

**FIGURE 2:**
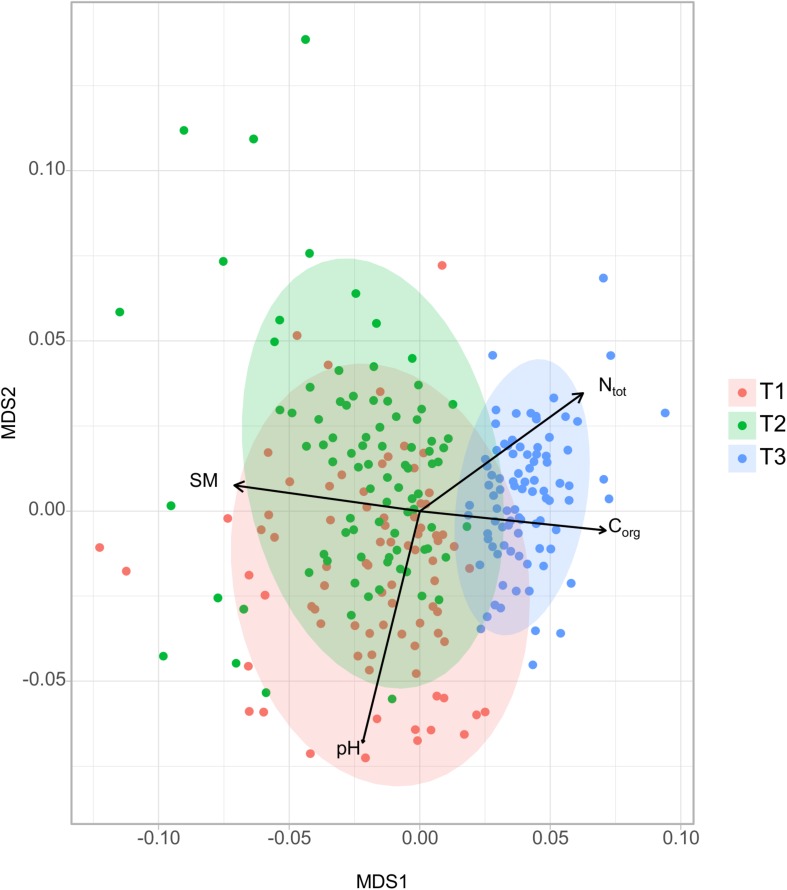
NMDS ordinations based on weighted unifrac distance matrix of soil bacterial community (stress value = 0.17). Ellipses show clustering of samples by sampling time. Significant explanatory variables are represented as arrows (PermANOVA, *P* < 0.001).

Non-metric multidimensional scaling analysis of soil bacterial community showed that dissimilarities in soil bacterial composition were mostly driven by sampling time (Figure 2). Bacterial community composition from soils collected at spring barley flowering (T3) were strongly different from soils collected in cover crop (T1) (pairwise-PermANOVA T3 vs. T1; *R*^2^ = 0.20, *P* = 0.001) or before the spring barley sowing (pairwise-PermANOVA T3 vs. T2; *R*^2^ = 0.19, *P* = 0.001). Significant but smaller differences in bacterial community composition were also observed between T1 and T2 (T1 vs. T2; *R*^2^ = 0.06, *P* = 0.001). To assess the relative importance of soil physico-chemical properties for bacterial community composition, significant soil properties (PermANOVA, *P* < 0.05) were selected and fitted to the ordination. Soil moisture (min = 7.14%, max = 26.42%) was the main factor explaining the differences in the bacterial community structure between sampling dates (Figure 2; *R*^2^ = 0.25, *P* < 0.001). These effects could be explained by seasonal patterns in precipitation and temperature, which have previously been described as important driver of temporal variability in soil microbial community structure (Rasche et al., 2011; Silva et al., 2012; Lauber et al., 2013). Indeed, soil moisture for example not only control oxygen diffusion but also nutrient availability, both being of high importance for microbial communities (Schimel, 2018). The bacterial community structure at T3 was also correlated to total soil N (*R*^2^ = 0.18, *P* < 0.001) and total soil organic C (*R*^2^ = 0.17, *P* < 0.001). Soil pH has been reported as an important determinant of bacterial communities (Fierer and Jackson, 2006; Carrera et al., 2007; Buyer et al., 2010; Rousk et al., 2010). Accordingly, the NMDS analysis shows that pH was a significant but a minor driver of changes in the community structure between T1 and T2 likely due to the small pH variability.

When calculating various α-diversity metric for the bacterial diversity, we also found significant correlation between total N or organic C and bacterial communities. Total N was negatively correlated to phylogenetic diversity, represented by Faith’s phylogenetic diversity independent of the sampling time, while organic C was negatively correlated to phylogenetic diversity at T2 only (Supplementary Figure S4 and Supplementary Table S4). Similarly, we found that total N had a negative impact on bacterial species richness. These results are consistent with previous studies showing a decrease in bacterial diversity in response to soil N additions (Campbell et al., 2010; Koyama et al., 2014; Zeng et al., 2016). In contrast other studies did not find any significant relationship between N-input and bacterial diversity (Leff et al., 2015). The abundance of the total bacterial community, which varied from 3.6 × 10^8^ to 3.3 × 10^9^ copy numbers per gram of dry soil within all treatments, was not affected by total N. However, total N was significantly positively correlated to the abundance of AOA and denitrifiers as quantified by qPCR assays using the nitrite reductase (*nirK*, *nirS*) and nitrous oxide reductase (*nosZI*) genes. This suggests that several N-cycling microbial communities are highly responsive to N dynamics during our experiment (Supplementary Table S5).

**FIGURE 3:**
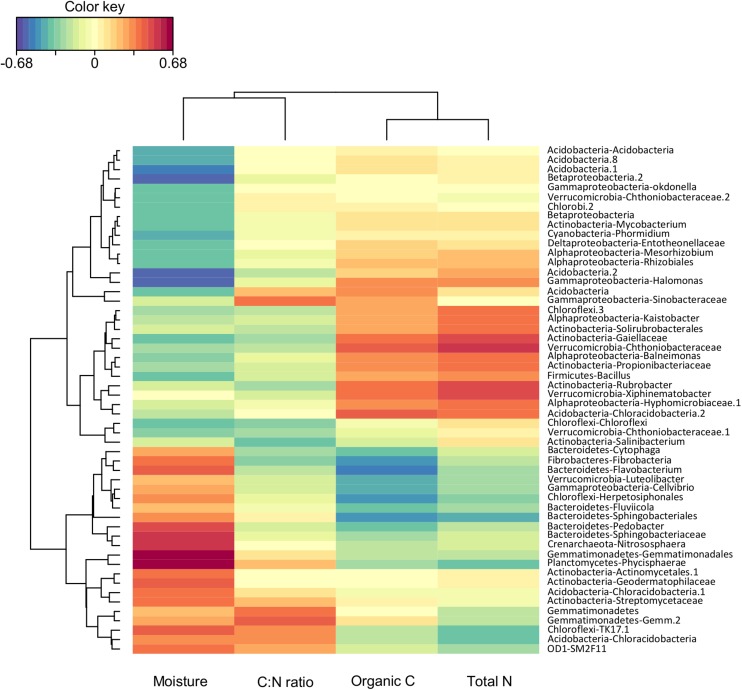
Heatmap plot representing correlation analysis between relative abundance of soil bacterial community at genus level and soil physico-chemical properties. Colors range from blue (low) to red (high) and represent negative to positive correlations, respectively.

The composition of soil bacterial community at the genus level was significantly affected by soil properties (i.e., pH, total N, organic C, and soil moisture; Supplementary Table S4). To identify bacterial groups that were significantly associated to one or more soil properties, we performed a rCCA between the proportion of the different bacterial genus and soil properties (Figure 3). Eighteen percent of the identified bacterial genera were strongly correlated to soil moisture, C:N ratio, and total N and organic C. Bacteria genera such as *Phycisphaerae* (*Planctomycetes*), *Nitrososphaera* (*Crenarchaeota*), *Sphingobacteriaceae*, and *Pedobacter* (*Bacteroidetes*) were positively correlated to soil moisture, while *Kaistobacter* (*Alphaproteobacteria*), *Solirubrobacterales*, *Gaiellaceae*, *Rubro- bacter* (*Actinobacteria*), *Bacillus* (*Firmicutes*), and *Xiphine- matobacter* (*Verrucomicrobia*) were positively correlated to total N and organic C (Figure 3). These results are in agreement with previous work showing shifts in the relative abundance of several bacterial groups in response to N addition (Ramirez et al., 2012). Similarly, the importance of soil moisture as a fundamental control over soil microbe survival and function has largely been described in the literature with differential responses to variation in soil water content between taxa depending on their physiology and life strategies (Schimel, 2018). Results of the CCA were consistent with the NMDS analysis indicating that soil properties such as moisture, total N, and organic C play a major role in structuring soil bacterial communities (Figure 2).

### Effects of Cover Crop Management on Bacterial Community Structure, Abundance, and Diversity

The studied cover crop management strategies strongly impacted soil properties (i.e., total N and organic C), which in turn had significant impacts on bacterial community structure, abundance, and diversity. Likewise, cover crop management strategies can also directly affect bacterial communities. Accordingly, we found significant modifications (PermANOVA, *P* < 0.001) in soil bacterial community structure for cover crop termination and irrigation treatments, while cover crops mixture had a minor, but still significant effect. Only soils with two non-legume cover crops differed from bare soil control (pairwise-PermANOVA, *P* = 0.015). At T1, significant differences in bacterial communities were observed between all termination treatments while at T2, only the glyphosate treatment differed significantly from the others. Finally, at T3 bacterial community in the rolling treatment was different from glyphosate treatment but similar to the frost one (pairwise-PermANOVA, *P* < 0.05). This could be due to the fact that cover crops were terminated at a later stage by glyphosate compared to the rolling and frost treatments, which can have many consequences for plant–microbe interactions. This was supported by the ANOVA model analysis (Supplementary Table S4) showing that sampling time had the major effect on bacterial genera relative abundance (97% of the detected genera), followed by the cover crop termination treatment (19%), irrigation (11%), and then cover crop mixture (1%). Only two bacterial groups belonging to *Solirubrobacterales* (*Actinobacteria*) and to *Ellin5301* (*Gemmatimonadales*) showed a significant response to cover crop mixture treatments with an increase in their relative proportion in the 2 Leg− cover crop mixture (i.e., cereal rye and phacelia) in comparison to the other treatments (ANOVA, *P* < 0.05). Interestingly, this cover crop mix showed the highest cover crop biomass, independent of the cover crop management (Supplementary Table S6).

**FIGURE 4:**
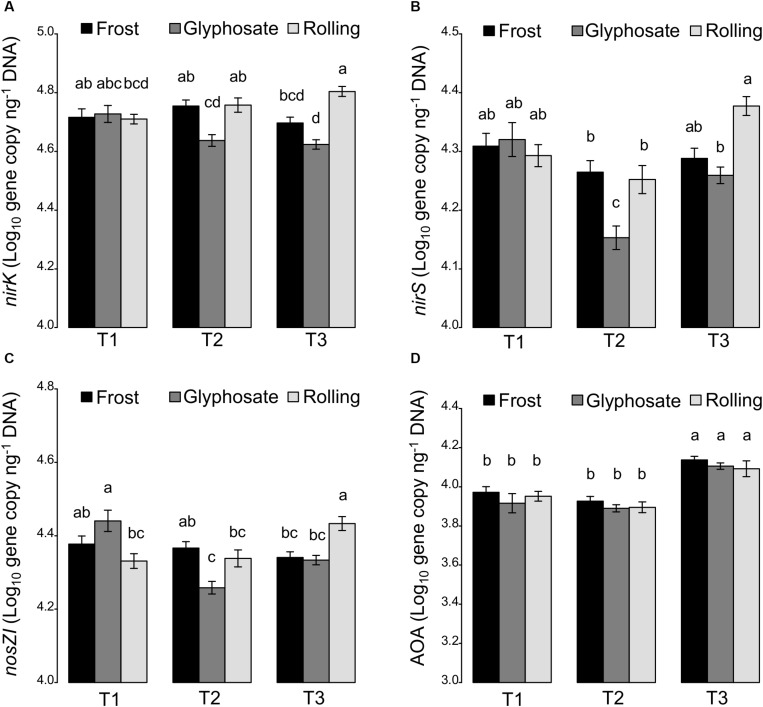
Significant effects of the interaction between cover crop termination (frost, glyphosate, and rolling) and sampling time (T1, T2, and T3) on the abundance of N cycling communities. **(A)** *nirK* denitrifiers, **(B)** *nirS* denitrifiers, **(C)** *nosZI* denitrifiers, and **(D)** ammonia oxidizing archaea (AOA) (Log_10_ gene copy ng^–1^ DNA). All values are means ± standard errors.

While cover crops have been reported to increase soil microbial biomass (Carrera et al., 2007; Buyer et al., 2010), we found that cover crops had no effect on the abundance of the total bacterial community as estimated by 16S rRNA real-time PCR quantification (Supplementary Table S5). However, abundance of bacterial denitrifiers (using the *nirK*, *nirS*, and *nosZI* genes as molecular markers) significantly decreased at T2 in glyphosate-terminated cover crops, while their abundance remained unchanged in frost-terminated cover crops, and even increased at T3 when cover crops were rolled (Figure 4). Such impact of the termination treatment on denitrifiers can be explained by the differential effects of termination treatments on total N (Figure 1A), with increased denitrifier abundance in treatments stimulating soil total N (i.e., rolling). Previous studies showed that cover crops could stimulate N-immobilization in the microbial biomass (In’t Zandt et al., 2018), which could have a detrimental effect on denitrifiers that are using inorganic N for respiration. Since an impact of glyphosate on soil microbial community has been reported in a recent meta-analysis (Nguyen et al., 2016), we cannot rule out a direct toxic effect on some microbial taxa involved in denitrification at our field site. In contrast, the termination method had little to no effect on ammonia oxidizers with only a significant but weak effect of the interaction of cover crop termination with sampling time.

The different α-diversity metrics for soil bacterial community were highly variable across the studied period. For example, both phylogenetic diversity and richness decreased by the end of spring barley crop, while the Simpson’s reciprocal index increased over time (ANOVA, *P* < 0.05, Supplementary Tables S4, S7). Similarly, Lauber et al. (2013) observed a significant temporal variability of the bacterial α-diversity, which exceeded the variability between land-use types. In our work it was not possible to decipher whether these temporal changes are directly related to the agricultural practices or to the seasonal variability affecting for examples soil moisture and temperature. However, we did find that the observed species index and the Simpson reciprocal index were significantly affected by the cover crops mixtures and cover crop termination treatments, respectively (Supplementary Table S4). Thus, soils with two non-legume cover species (i.e., 2 Leg−, cereal rye and phacelia), which also produced the highest cover crop biomass, exhibited a higher bacterial species richness than the soils with eight legume cover species (ANOVA, *P* < 0.05, Supplementary Table S4 and Supplementary Figure S5). This is in agreement with previous study describing species-specific relationships between cover crops mixture and soil microbial communities due to differences in biomass production, in taxonomic (species richness) or functional (legumes vs. non-legumes) diversity in the cover crop mixture (Finney et al., 2017). This is also supported by a large body of literature showing significant effects of the plant species identity on microbial communities in the rhizosphere (reviewed in Philippot et al., 2013). Altogether, our work showed a significant effect of cover crops, which modified the diversity of soil bacterial communities. However, while a positive relationship between above- and below-ground diversity has been previously reported by several studies (Wardle et al., 2004; De Deyn and Van der Putten, 2005), here we didn’t observe such link between the diversity of cover crops (neither taxonomic nor functional) and of bacterial communities.

## Conclusion

Overall, our results showed that cover crop management practices are more important drivers of both composition of the total bacterial community and abundance of N-cycling microbial guilds than cover crop mixtures. Frost-terminated cover crops released N at crop flowering while rolled cover crops showed a constant increase in total N content from the cover crop period to the succeeding crop flowering stage. We also showed that the abundance of denitrifiers increased in the rolling treatment while in contrast glyphosate treatment resulted in lower denitrifier abundance. Within the N cycle, denitrifiers are responsible for N losses to the atmosphere, therefore reducing available N for crop. Given the higher levels of total N and organic C in soils when cover crops were destroyed by frost, this termination method could be proposed as an efficient strategy for enhancing nutrient cycling in agricultural soils. However, accounting only on frost to winter-kill cover crops is considered as a risky strategy (Parr et al., 2014), while farmers are facing unforeseeable weather conditions (Wayman et al., 2017). Further research are therefore needed to investigate effects of cover cropping in long-term field experiments with a wider range of cover crop mixtures, in order to identify management strategies that can be integrated into sustainable agricultural systems.

## Author Contributions

AS and SC contributed to the conception and design of the study. SR, LP, AS, and SC wrote the manuscript. SR performed the statistical analysis. HB, LF, JM, FB, DB, and M-CB performed the soil sampling, and soil and DNA analysis. All authors read and approved the submitted manuscript.

## Conflict of Interest Statement

The authors declare that the research was conducted in the absence of any commercial or financial relationships that could be construed as a potential conflict of interest.
