# Supplementary material for: Cover Crop Management Practices Rather Than Composition of Cover Crop Mixtures Affect Bacterial Communities in No-Till Agroecosystems
Source: Front Microbiol. 2019 Jul 9;10:1618. doi: 10.3389/fmicb.2019.01618 (PMC6629898; doi:10.3389/fmicb.2019.01618)
Supplement: Supplementary file 1 [file Data_Sheet_1.PDF]

Supplementary Material:

Cover Crop Management Practices Rather Than Composition of Cover Crop Mixtures Affect Bacterial Community in No-Till Agroecosystems

Sana Romdhane[1*]. Aymé Spor[1*]. Hugues Busset[1]. Laurent Falchetto[2]. Juliette Martin[2]. Florian Bizouard[1]. David Bru[1]. Marie-Christine Breuil[1]. Laurent Philippot[1] and Stéphane Cordeau[1]

[1] *Agroécologie. AgroSup Dijon. INRA. Univ. Bourgogne Franche-Comté. F-21000 Dijon. France*
[2] *INRA. UE115 Domaine Expérimental d'Epoisses. F-21000 Dijon. France*
[*] *Correspondences: Dr. Sana Romdhane. sanaromdhan@gmail.com and Dr. Aymé Spor. ayme.spor@inra.fr.*

Table S1: Plant species identity in the Cover crop mixtures including 12 different species with and without legumes: 2 species without legume (2 Leg-). 2 species with legumes (2 Leg+). 8 species without legumes (8 Leg-) and 8 species with legumes (8 Leg+).

		2 Leg -	2 Leg +	8 Leg -	8 Leg+
Grasses	Bristle Oat		X	X	
	Cereal rye	X		X	X
Legumes	Vetch		X		X
	Berseem clover				X
	Spring faba bean				X
	Sunn hemp				X
Brassica	Brown mustard			X	X
	Wild turnip			X	X
Others	Lacy phacelia	X		X	
	Niger			X	X
	Buckwheat			X	
	Spring flaxseed			X	

Table S2: Soil properties means at different sampling times within all treatments. Soil moisture is expressed as a percentage. Organic carbon, organic matter and total nitrogen are expressed as g kg^{-1} dry soil.

ST	Soil moisture	Standard Error	Min	Max
**T1**	18.51	0.32	11.98	25.31
**T2**	18.89	0.40	10.72	26.42
**T3**	13.66	0.36	7.14	20.89
	**pH**	**Standard Error**	**Min**	**Max**
**T1**	7.17	0.18	7.00	7.40
**T2**	7.07	0.01	6.90	7.30
**T3**	7.07	0.01	6.80	7.30
	**Organic Carbon**	**Standard Error**	**Min**	**Max**
**T1**	15.35	0.50	13.00	20.00
**T2**	14.16	0.08	12.20	15.80
**T3**	15.59	0.10	13.10	17.70
	**Organic Matter**	**Standard Error**	**Min**	**Max**
**T1**	26.55	0.87	22.40	34.60
**T2**	24.49	0.14	21.10	27.20
**T3**	26.96	0.16	22.70	30.60
	**Total Nitrogen**	**Standard Error**	**Min**	**Max**
**T1**	1.40	0.04	1.11	1.57
**T2**	1.40	0.01	1.13	1.59
**T3**	1.48	0.01	1.23	1.79
	**C:N ratio**	**Standard Error**	**Min**	**Max**
**T1**	10.52	0.34	10.00	13.00
**T2**	9.92	0.03	9.40	11.00
**T3**	10.09	0.04	9.20	12.00

Table S3: Results from ANOVA analysis of the effects of agricultural practices on soil physico-chemical properties. Only variables and factors with significant effects are shown. ST: Sampling Time. B: Block. CC: Cover crop mixture. CCT: Cover crop termination. IR: Irrigation level.

	**Total Nitrogen**			**Organic Carbon**			**C:N ratio**			**pH**			**Soil moisture**		
Agricultural practices	*Df*	*F*	*P*	*Df*	*F*	*P*	*Df*	*F*	*P*	*Df*	*F*	*P*	*Df*	*F*	*P*
ST	2	149.70	<0.05	2	153.68	<0.05	2	49.68	<0.05	2	69.83	<0.05	2	81.33	<0.05
Y position	1	1569.60	<0.05	1	300.86	<0.05	1	28.01	<0.05	1	27.94	<0.05	1	5.58	<0.05
B	2	18.47	<0.05	2	0.49	NS	2	7.70	<0.05	2	22.63	<0.05	2	1.62	NS
IR	1	22.26	NS	1	1.91	NS	1	0.56	NS	1	87.94	<0.05	1	1.83	NS
CCT	2	154.47	<0.05	2	58.87	<0.05	2	1.13	NS	2	5.18	<0.05	2	4.71	<0.05
CCT x CC	8	2.36	<0.05	8	0.31	NS	8	2.41	NS	8	1.01	NS	8	0.94	NS
ST x B	4	0.88	<0.05	4	3.48	<0.05	4	5.42	<0.05	4	0.32	NS	4	1.59	NS
ST x Y position	2	4.00	<0.05	2	0.40	NS	2	1.76	NS	2	7.38	<0.05	2	3.44	<0.05
ST x CCT	4	40.46	<0.05	4	11.63	<0.05	4	2.41	NS	4	1.28	NS	4	13.72	<0.05
B x CCT	6	7.22	<0.05	6	22.78	<0.05	6	0.84	NS	6	5.61	<0.05	6	0.11	NS
ST x B x CCT	8	6.60	<0.05	8	4.24	<0.05	8	2.56	<0.05	8	1.88	NS	8	1.39	<0.05
B x IR	3	30.42	<0.05	3	7.81	<0.05	3	0.06	NS	3	1.40	<0.05	3	0.41	NS

Table S4: Results from ANOVA analysis of the effects of soil properties and agricultural practices on soil bacterial community diversity (PD: PD Whole Tree, Simpson R: Simpson Reciprocal index and Richness: observed species) and abundances of bacterial groups at genus level (283 genera). Only variables and factors with significant effects are shown. ST: Sampling Time. N_{tot}: total nitrogen. SM: Soil moisture. M_{org}: Organic matter. B: Block. CC: Cover crop mixture. CCT: Cover crop termination. IR: Irrigation level.

	**PD**			**Simpson R**			**Richness**			**Abundances**		
Agricultural practices And soil proprieties	*df*	*F*	*P*	*df*	*F*	*P*	*df*	*F*	*P*	*df*	*P **	*% Genus*
ST	2	22.18	<0.05	2	8.58	<0.05	2	40.69	<0.05	2	<0.05	97 % (274)
N_{tot}	1	13.08	<0.05	1	0.65	NS	1	9.51	<0.05	1	<0.05	25 % (72)
pH	1	0.02	NS	1	2.33	NS	1	0.55	NS	1	<0.05	30 % (85)
SM	1	0.92	NS	1	0.06	NS	1	0.33	NS	1	<0.05	14 % (40)
ST x N_{tot}	2	0.14	NS	2	1.95	NS	2	0.09	NS	2	<0.05	4 % (11)
ST x SM	2	0.88	NS	2	1.29	NS	2	0.09	NS	2	<0.05	19% (54)
ST x M_{org}	3	3.51	NS	3	1.68	NS	3	3.64	<0.05	3	<0.05	0.7 % (2)
ST x C_{org}	3	2.65	0.05	3	0.92	NS	3	1.39	NS	3	<0.05	0.7 % (2)
B	2	3.54	<0.05	2	2.59	NS	2	2.46	NS	2	<0.05	11 % (30)
CC	4	2.22	NS	4	1.68	NS	4	3.65	<0.05	4	<0.05	1 % (2)
CCT	2	0.88	NS	2	4.43	<0.05	2	0.25	NS	2	<0.05	19 % (55)
IR	1	0.52	NS	1	3.61	NS	1	1.88	NS	1	<0.05	10 % (28)
ST x B	6	2.98	<0.05	6	0.85	NS	6	2.32	NS	6	<0.05	6 % (57)
ST x CC	8	1.07	NS	8	0.77	NS	8	0.86	NS	8	<0.05	0.7 % (2)
ST x CCT x CC	20	1.10	NS	20	1.45	NS	20	1.00	NS	20	<0.05	1 % (3)
ST x B x IR	6	2.25	<0.05	6	0.76	NS	6	0.15	NS	6	NS	-
B x IR	2	0.62	NS	2	0.81	NS	2	0.79	NS	2	<0.05	1.8 % (5)
B x CCT	4	0.06	NS	4	0.57	NS	4	0.28	NS	4	<0.05	1.8 % (5)
CCT x CC	8	0.19	NS	8	0.31	NS	8	0.28	NS	8	<0.05	0.5 % (1)

Table S5: Results from ANOVA analysis of the effects of soil properties and agricultural practices on the abundances of total bacteria, archaea, nitrifiers and denitrifiers (Log_{10} gene copy g^{-1} DNA). ST: Sampling Time. N_{tot}: total nitrogen. SM: Soil moisture. M_{org}: Organic matter. B: Block. CC: Cover crop mixture. CCT: Cover crop termination. IR: Irrigation level.

	**16S**			**crena**			**nir*K***			**nir*S***		
Agricultural practices And soil proprieties	*Df*	*F*	*P*	*Df*	*F*	*P*	*Df*	*F*	*P*	*Df*	*F*	*P*
ST	2	53.39	<0.05	2	66.09	<0.05	2	0.15	ns	2	22.74	<0.05
N_{tot}	1	0.03	NS	1	22.52	<0.05	1	13.36	<0.05	1	5.40	<0.05
pH	1	0.92	NS	1	0.80	NS	1	6.59	<0.05	1	4.23	<0.05
ST x N_{tot}	2	1.39	NS	2	0.004	NS	2	5.81	<0.05	2	3.21	NS
ST x SM	3	2.75	<0.05	3	1.28	NS	3	3.58	<0.05	3	2.14	NS
ST x C:N	2	1.52	NS	2	0.33	NS	2	1.35	NS	2	0.80	NS
ST x C_{org}	3	3.01	<0.05	3	1.04	NS	3	4.46	<0.05	3	5.65	<0.05
ST x pH	2	0.77	NS	2	0.79	NS	2	3.90	<0.05	2	1.70	NS
B	2	0.55	NS	2	0.01	NS	2	11.24	<0.05	2	3.60	<0.05
CC	4	0.70	NS	4	1.79	NS	4	1.84	NS	4	3.26	<0.05
CCT	2	2.81	NS	2	0.11	NS	2	24.66	<0.05	2	14.38	<0.05
IR	1	1.54	NS	1	0.27	NS	1	4.01	<0.05	1	1.10	NS
ST x B	4	6.51	<0.05	4	3.24	<0.05	4	5.28	<0.05	4	5.00	<0.05
ST x CCT	4	1.82	NS	4	0.27	NS	4	3.74	<0.05	4	2.44	<0.05
ST x IR	2	2.47	NS	2	1.07	NS	2	1.70	NS	2	3.42	<0.05
ST x B x CCT	8	0.89	NS	8	1.57	NS	8	1.21	NS	8	1.35	NS
ST x B x IR	4	2.93	<0.05	4	1.69	NS	4	3.26	<0.05	4	1.60	NS
ST x CC x IR	16	0.67	NS	16	0.39	NS	16	1.36	NS	16	1.37	NS
B x IR	2	0.81	NS	2	0.10	NS	2	2.00	NS	2	3.54	<0.05
B x CCT	4	3.78	<0.05	4	5.06	<0.05	4	3.34	<0.05	4	3.94	<0.05
CC x CCT	8	1.37	NS	8	1.79	NS	8	1.08	NS	8		<0.05
IR x CC	4	0.39	NS	4	0.96	NS	4	1.35	NS	4	2.91	<0.05
IR x CCT	2	0.97	NS	2	0.40	NS	2	3.29	<0.05	2	5.00	<0.05
IR x CC x CCT	8	1.32	NS	8	1.46	NS	8	1.95	NS	8	2.96	<0.05

	nos*Z1*			nos*Z2*			AOA			AOB		
Agricultural practices And soil proprieties	*Df*	*F*	*P*	*Df*	*F*	*P*	*Df*	*F*	*P*	*Df*	*F*	*P*
ST	2	8.85	<0.05	2	24.33	<0.05	2	63.76	<0.05	2	16.38	<0.05
N_{tot}	1	4.08	<0.05	1	0.50	NS	1	65.61	<0.05	1	0.53	NS
pH	1	0.47	NS	1	2.66	NS	1	14.88	<0.05	1	8.90	<0.05
ST x N_{tot}	2	2.29	NS	2	0.65	NS	2	2.06	NS	2	1.91	NS
ST x SM	3	2.03	NS	3	8.13	<0.05	3	0.38	NS	3	2.33	NS
ST x C:N	2	0.49	NS	2	0.27	NS	2	1.14	NS	2	5.22	<0.05
ST x C_{org}	3	8.55	<0.05	3	3.13	<0.05	3	1.27	NS	3	1.65	NS
ST x pH	2	0.14	NS	2	0.25	NS	2	3.18	<0.05	2	1.73	NS
B	2	2.50	NS	2	0.02	NS	2	6.62	<0.05	2	0.28	NS
CC	4	1.16	NS	4	1.77	NS	4	1.51	NS	4	2.04	NS
CCT	2	3.27	<0.05	2	0.41	NS	2	1.42	NS	2	1.31	NS
IR	1	6.20	<0.05	1	2.10	NS	1	2.00	NS	1	10.18	<0.05
ST x B	4	10.25	<0.05	4	4.43	<0.05	4	0.32	NS	4	1.78	NS
ST x CCT	4	3.31	<0.05	4	1.10	NS	4	3.42	<0.05	4	0.68	NS
ST x IR	2	4.68	<0.05	2	1.14	NS	2	1.35		2	1.84	NS
ST x B x CCT	8	0.70	NS	8	1.02	NS	8	2.71	<0.05	8	2.92	<0.05
ST x B x IR	4	1.60	NS	4	2.51	<0.05	4	0.97	NS	4	2.75	<0.05
ST x CC x IR	16	1.15	NS	16	1.57	NS	16	0.86	NS	16	2.01	<0.05
B x IR	2	0.43	NS	2	1.06	NS	2	0.32	NS	2	5.20	<0.05
B x CCT	4	3.10	<0.05	4	3.42	<0.05	4	0.23	NS	4	0.42	NS
CC x CCT	8	2.05	<0.05	8	1.11	NS	8	0.67	NS	8	1.84	NS
IR x CC	4	1.17	NS	4	0.60	NS	4		NS	4	2.88	<0.05
IR x CCT	2	2.53	NS	2	3.33	<0.05	2	3.22	<0.05	2	0.87	NS
IR x CC x CCT	8	1.78	NS	8	1.60	NS	8	1.23	NS	8	1.53	NS

Table S6: Cover crop biomass means measured at T1 for different cover crop modalities (g dry mass/m²).

ST	Biomass	Standard Error	Min	Max
**2 Leg-**	228.02	81.11	95.40	382.80
**2 Leg+**	131.48	35.46	58.00	196.80
**8 Leg-**	145.09	21.95	97.30	182.10
**8 Leg+**	146.30	26.02	98.90	194.20

Table S7: Means of α-diversity metrics of the soil bacterial community at different sampling times within all treatments (Richness: observed species, PD: PD Whole Tree and Simpson R: Simpson Reciprocal index).

ST	Richness	Standard Error	Min	Max
**T1**	1603.91	52.91	1470	1738
**T2**	1626.77	64.55	1334	1724
**T3**	1556.55	44.74	1408	1663
	**PD**	**Standard Error**	**Min**	**Max**
**T1**	102.87	2.95	97.29	110.36
**T2**	103.42	3.73	87.78	110.16
**T3**	100.45	3.19	87.71	108.79
	**Simpson R**	**Standard Error**	**Min**	**Max**
**T1**	240.78	36.02	125.01	316.25
**T2**	246.39	55.47	36.55	348.79
**T3**	266.34	38.61	69.11	321.66

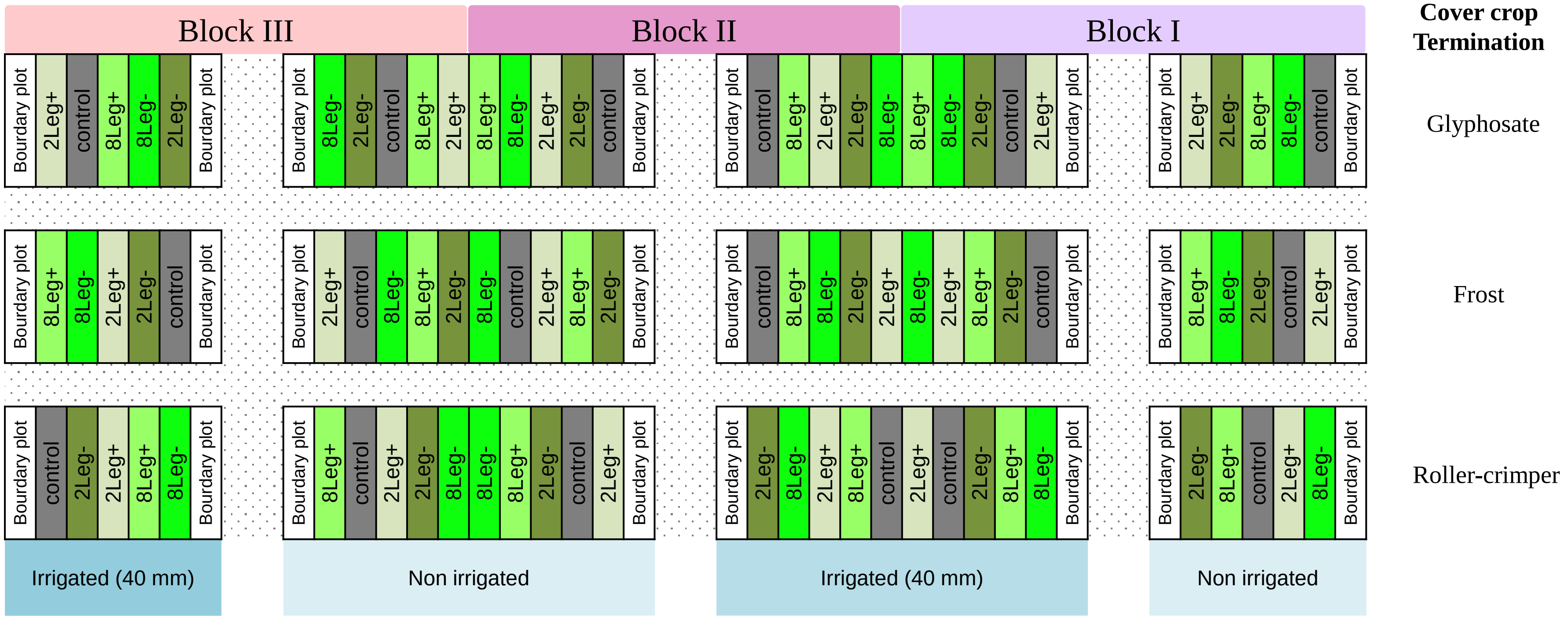

Figure S1: Experimental design of the cover crop experiment.

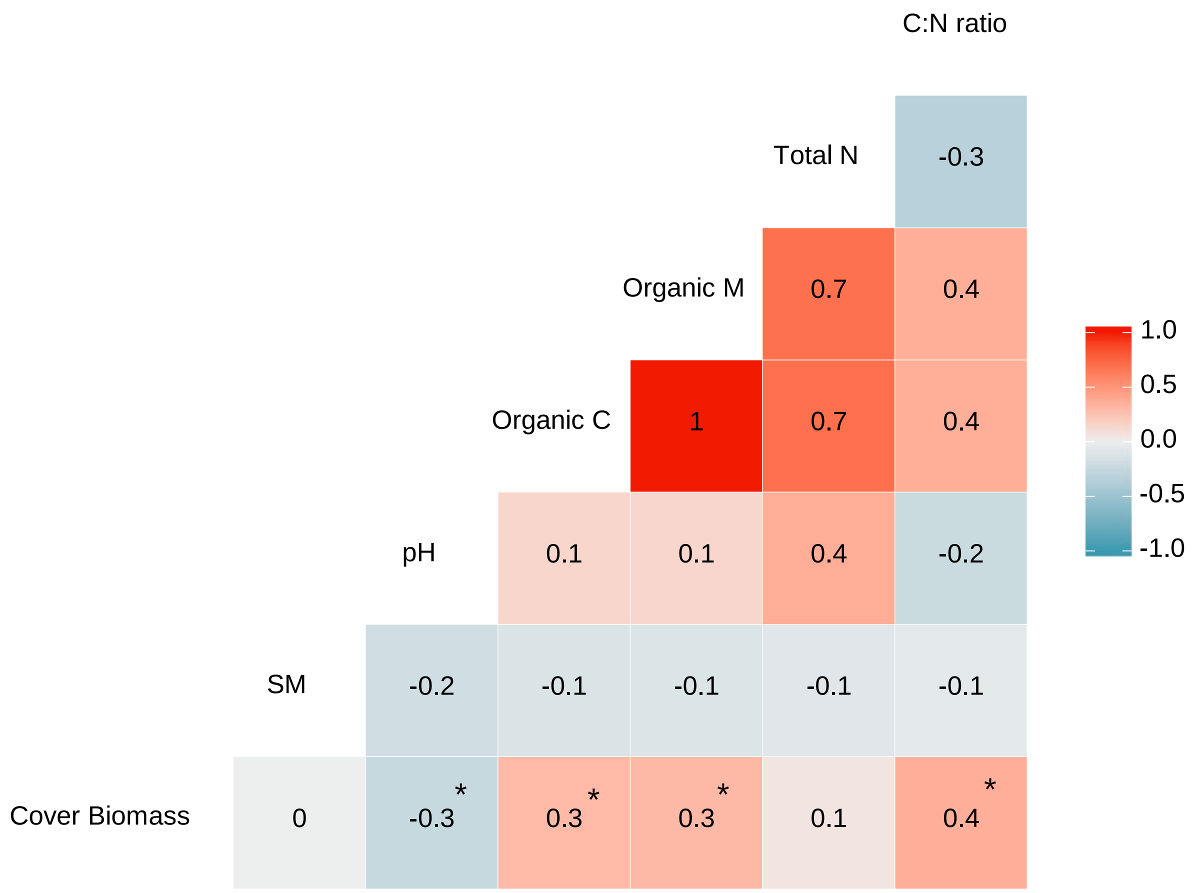

Figure S2. Pearson correlation analysis between the cover biomass and soil properties. SM: Soil moisture. Organic C: Organic carbon. Organic M: Organic matter. Total N: total nitrogen. Red represents positive correlations and blue represents negative correlations.

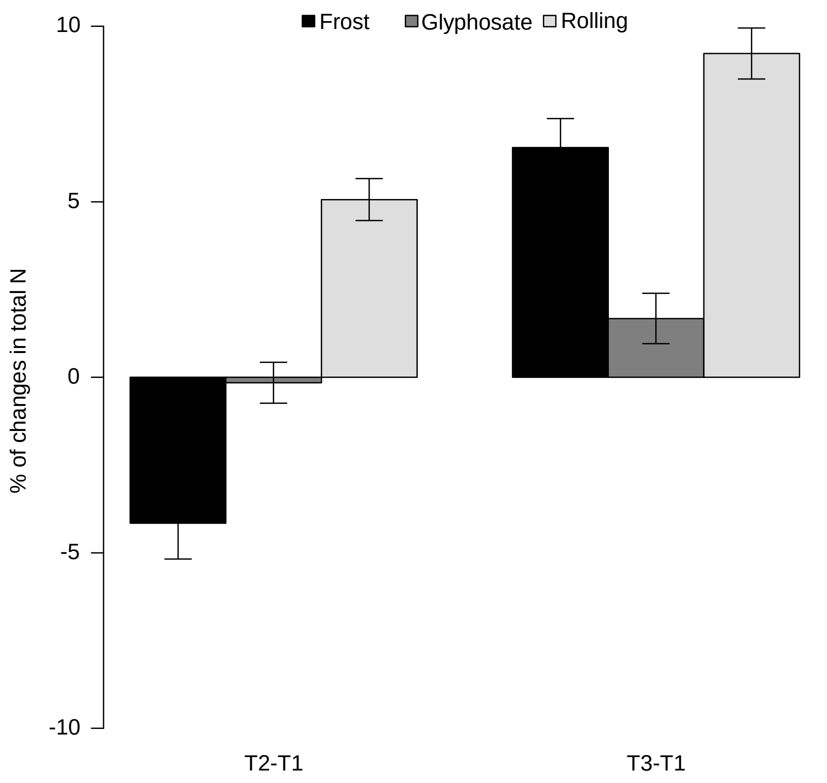

Figure S3. Percentage of changes in total N as measured between sampling times T2 and T1 as well as T3 and T1 for different termination methods.

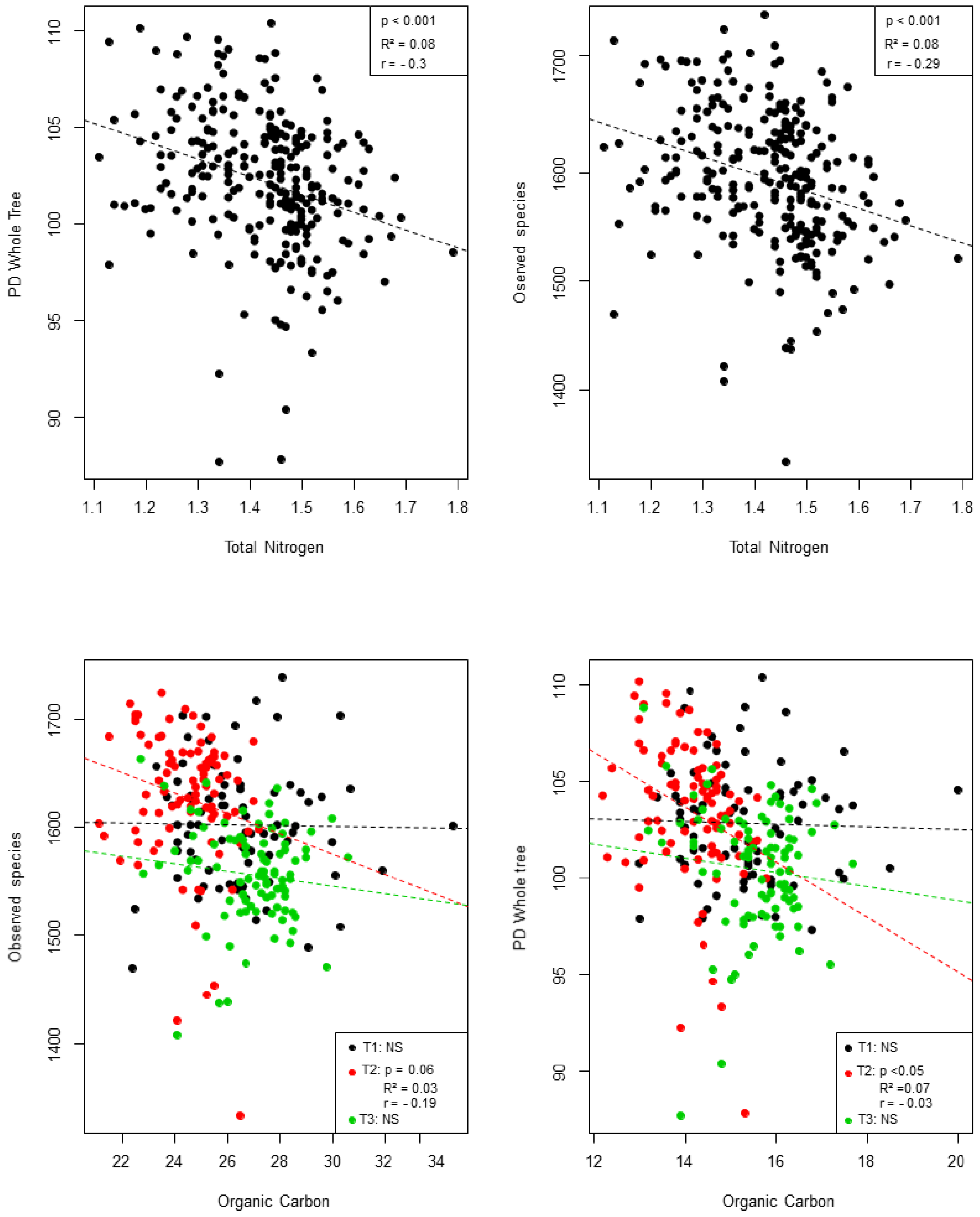

Figure S4: Effects of soil properties on soil bacterial diversity as estimated by PD whole tree and observed species indices.

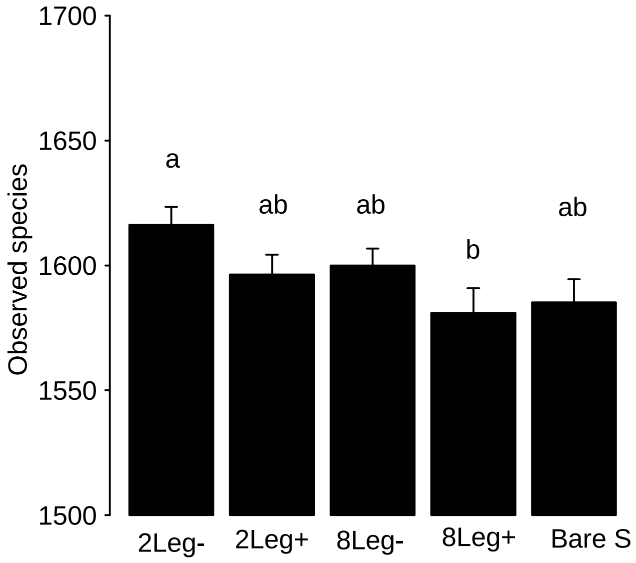

Figure S5: Effects of cover crop mixtures on soil bacterial richness as estimated by observed species index.
